# Rationale and design of the HINODE study: Heart failure indication and sudden cardiac death prevention trial Japan

**DOI:** 10.1002/joa3.12577

**Published:** 2021-07-20

**Authors:** Hiro Yamasaki, Kenji Ando, Takanori Ikeda, Takeshi Mitsuhashi, Toyoaki Murohara, Nobuhiro Nishii, Akihiko Nogami, Yasushi Sakata, Wataru Shimizu, Torri Simon, Caroline Beaudoint, Torsten Kayser, Valentina Kutyifa, Kazutaka Aonuma

**Affiliations:** ^1^ Department of Cardiology Faculty of Medicine University of Tsukuba Ibaraki Japan; ^2^ Department of Cardiology Kokura Memorial Hospital Fukuoka Japan; ^3^ Department of Cardiovascular Medicine Faculty of Medicine Toho University Tokyo Japan; ^4^ Department of Cardiology Hoshi General Hospital Fukushima Japan; ^5^ Department of Cardiology Nagoya University Graduate School of Medicine Aichi Japan; ^6^ Department of Cardiovascular Therapeutics Okayama University Graduate School of Medicine, Dentistry, and Pharmaceutical Sciences Okayama Japan; ^7^ Department of Cardiovascular Medicine Osaka University Graduate School of Medicine Osaka Japan; ^8^ Department of Cardiovascular Medicine Nippon Medical School Tokyo Japan; ^9^ Department of Biostatistics Boston Scientific St Paul MN USA; ^10^ Department Clinical Research Boston Scientific Diegem Belgium; ^11^ University of Rochester Rochester NY USA

**Keywords:** defibrillator therapy, heart failure, Japan, resynchronization therapy, sudden cardiac death

## Abstract

**Background:**

Randomized trials in Western countries have provided evidence that prophylactic implantable cardioverter‐defibrillator (ICD) therapy reduces mortality in heart failure (HF) patients with reduced left ventricular ejection fraction. However, the risk of life‐threatening ventricular arrhythmias in Japanese HF patients sharing similar risk factors is still unknown.

**Methods:**

The Heart Failure Indication and Sudden Cardiac Death Prevention Trial Japan trial (NCT03185832) is a prospective, multicenter registry designed to collect data on ventricular arrhythmia, HF events, and mortality in Japanese HF patients. Japanese patients with HF and 2‐5 predefined risk factors who were indicated for cardiac device implantation based on European Society of Cardiology guidelines were enrolled in four treatment arms: implantable cardioverter‐defibrillator (ICD), cardiac resynchronization therapy defibrillator (CRT‐D), HF pacing (PA; Pacemaker and cardiac resynchronization pacemaker), and nondevice (ND) cohorts and followed for a minimum of 12 months. Since it is anticipated that some baseline patient characteristics and risk factors will differ significantly from those reported in predominantly Western populations, event rates will be compared to a propensity‐matched population from the MADIT RIT trial. Primary endpoints are composite rates of first appropriately treated ventricular arrhythmias (VA) or/and life‐threatening VA symptoms for the ICD and CRT‐D cohorts. For nondevice and PA cohorts, the primary outcome is all‐cause mortality.

**Conclusions:**

The Heart Failure Indication and Sudden Cardiac Death Prevention Trial Japan is a large prospective multicenter registry with defined device treatment cohorts and will provide data for risk stratification for cardiovascular events in Japanese HF patients.

## 1 INTRODUCTION

The prevalence of chronic heart failure (HF) is expected to rapidly increase in the next decades in many industrialized countries, including Japan.^1^ Large cohort studies are useful for risk stratification and determination of preventive measures for this disorder.^2^ Notably, large randomized trials have shown the benefit of implantable cardioverter‐defibrillator (ICD) and cardiac resynchronization therapy‐defibrillator (CRT‐D) therapy for specific HF patients,^3^, ^4^, ^5^ and the results have been included in treatment guidelines.^6^ However, those studies primarily enrolled patients from the United States (US) and Europe and may not generalize to Japan because of genetic and cultural differences. Indeed, retrospective^7^ and prospective registry^8^ studies conducted in Japan have yielded contradictory results regarding the rate of sudden cardiac death (SCD) and need for ICD therapy for primary prevention in Japanese patients. Early studies found lower rates of life‐threatening ventricular arrhythmias^9^ while more recent Japanese studies using the European Society of Cardiology (ESC) guidelines for classification of patient groups^10^ or Multicenter Automatic Defibrillator Implantation Trial (MADIT) II‐like criteria,^11^ have suggested a benefit to primary prevention ICD implantation in this population. Because of the lack of clear evidence, device utilization in Japan varies by implant center and physician. Large device treatment registries (eg Nippon Storm) are already initiated for better therapy understanding in Japan and collect a wide range of clinical background data,^12^ but a large‐scale prospective study with Japanese patients is needed to clarify effectiveness of primary prevention ICD therapy for Japanese patients with HF.

The Heart Failure Indication and Sudden Cardiac Death Prevention Trial Japan (HINODE) prospective cohort trial was designed to identify patients with highest risk for SCD and HF events and compare event rates to those observed in predominantly Western populations. The study is examining outcomes across four treatment groups: implantable cardioverter‐defibrillator (ICD), cardiac resynchronization therapy defibrillator (CRT‐D), nondevice (ND), and HF pacing (PA) cohort (including patients with CRT‐P devices).

## 2 METHODS

### 2.1 Study design

The HINODE trial (NCT03185832) is a prospective, multicenter registry designed to collect data on VA, HF events, and mortality in Japanese patients with HF. Forty centers were nominated by the steering committee, and site selection was based on an independent process, which took trial experience and subject availability into account. This study was conducted in accordance with the relevant parts of the International Conference on Harmonization Guidelines for GCP and the ethical principles of the Declaration of Helsinki. All centers were asked to follow the Japanese Ethics Guideline for Clinical Research on Human Subjects issued by the Ministry of Health, Labour and Welfare. Study centers were opened for enrollment after obtaining approval from the ethics committee (EC). Informed consent was mandatory, and all site‐specific adjustments were in accordance with the principles of the Declaration of Helsinki and ISO 14 155:2011 and accepted and approved by the site's EC or central EC. Adverse event classification and reporting followed the definitions of ISO 14 155:2011 and MEDDEV 2.7/4. To support data reporting, especially on endpoint‐relevant events, data monitoring at study sites was conducted regularly.

### 2.2 Characterization of study cohorts

Patients who underwent de novo implantation of ICD, CRT‐D, pacemaker (PM), or CRT pacing (CRT‐P) device within 45 days were screened for eligibility and enrollment. Screening was performed and informed consent for the participation of the study was obtained only after device implantation, and the decision of device implantation was solely determined by the investigator. Based on 2016 European Society of Cardiology (ESC) Guidelines for the diagnosis and treatment of acute and chronic heart failure,^6^ patients were included in the ICD cohort as long as they met the above entry criteria and had any ICD (transvenous or subcutaneous) implanted, as well as reduced LVEF (≤35%), NYHA class II or III functional status, and were prescribed guideline directed medical therapy^13^for ≥3 months. Likewise, patients were included into the CRT‐D cohort if they had any CRT‐D device implanted as long as they met the above criteria and had reduced LVEF (≤35%) and LBBB with QRS >130 ms or non‐LBBB with QRS ≥150 ms despite optimal medical therapy (OMT) or alternatively were in AF with NYHA Class III, and a QRS duration ≥130 ms despite optimal medical therapy (OMT). ECGs collected up to 45 days prior to device implant and post implant were used to verify CRT‐D enrollment criteria. Patients with a history of previous PM/ ICD/ CRT‐P/ CRT‐D were excluded from ICD/ CRT‐D cohorts. In the PA cohort, the enrollment criteria were: expectation of >40% right ventricular pacing, reduced LVEF ≤50%, QRS >90 ms, and any previous HF admission to the hospital. Enrolled nondevice (ND) patients met ICD or CRT‐D implantation criteria but were not implanted with a cardiac device. To control for the effects of risk factors on patient outcome,^14^, ^15^, ^16^, ^17^, ^18^ enrollment was restricted based on risk factors. Enrollment was limited to patients with 2 to 5 of the following risk factors: (a) left ventricular ejection fraction (LVEF) ≤35%, (b) New York Heart Association (NYHA) functional Class III or IV, (c) left bundle branch block (LBBB) with QRS ≥130 ms or any QRS morphology ≥150 ms, (d) renal dysfunction (chronic blood urea nitrogen [BUN] >26 mg/dL or ≥9.28 mmol/L), (e) diabetes type I and II, (f) chronic atrial fibrillation, (g) prior myocardial infarction, (h) age >70 years, or (i) smoking currently or during the last 5 years,^3^, ^4^, ^11^, ^14^, ^15^, ^16^, ^17^ see Table 1. Patients were excluded if they had chronic renal failure with BUN ≥50 mg/dL or creatinine ≥2.5 mg/dL, cardiac bypass surgery or percutaneous coronary intervention within 3 months, recent myocardial infarction with elevation of cardiac enzyme, medical conditions that would limit study participation (per physician discretion), pregnant, a life expectancy of less than 1 year, or device implantation from another manufacturer. An overview of the main inclusion and exclusion criteria is listed in Table 1.

**TABLE 1:** Overview of main inclusion and exclusion criteria

Main Criteria for Cohort Inclusion at Enrollment			
ICD Cohort	CRT‐D Cohort	Nondevice Cohort	Pacing Cohort
NYHA Class II–III	NYHA class I‐IV	Fulfilling criteria for either ICD or CRT‐D cohort	NYHA I – IV (Symptoms of HF, any previous HF admission, IV treatment, or upgrade to CRT‐P)
Ischemic heart disease or dilated cardiomyopathy	Sinus rhythm QRS ≥130ms + LBBB; QRS ≥150ms + non‐LBBB or AF rhythm (only NYHA class III) QRS ≥130ms		Estimated RV pacing >40% paced or intrinsic QRS >90ms
LVEF ≤35%			LVEF ≤50%
2‐5 risk factors out of: LVEF ≤35%, NYHA Class III or IV, LBBB with QRS ≥130ms or non‐LBBB QRS ≥150ms, renal dysfunction (BUN >26mg/dL), diabetes type I and II, chronic atrial fibrillation, prior myocardial infarction, age >70 years, and smoking currently or during last 5 years			
General main exclusion criteria and cohort‐specific exclusions			
More than five risk factors, chronic renal failure (chronic BUN ≥50 mg/dL or creatinine ≥2.5 mg/dL), cardiac bypass surgery or percutaneous coronary intervention within 3 months, or recent myocardial infarction with the elevation of cardiac enzyme, a life expectancy of less than 1 year Any previous PM/ICD/CRT‐P/CRT‐D except upgrades from PM to CRT‐P in the Pacing cohort.			

Note

BUN, blood urea nitrogen; CRT‐D, cardiac resynchronization therapy defibrillator; CRT‐P, cardiac resynchronization therapy pacemaker; HF, heart failure; ICD, implantable cardioverter defibrillator; LBBB, left bundle branch block; LVEF, left ventricular ejection fraction; NYHA, New York Heart Association; PM, pacemaker; RV, right ventricular; VA, ventricular arrhythmia.

After obtaining informed consent, defibrillator programming was adjusted based on MADIT RIT arm B or C for transvenous ICDs and CRT‐Ds^19^or the UNTOUCHED^20^study for S‐ICDs (Table 2). MADIT RIT demonstrated in ICD and CRT‐D indicated primary prevention patients that first inappropriate shocks could be reduced by 75% while programming a therapy cut‐off rate ≥200 bpm (arm B) or a 60s delayed therapy at a heart rate ≥170 bpm (arm C).^21^ The UNTOUCHED study confirmed the effect of high rate cut‐off programming on resulting in a low rate of appropriate and inappropriate therapy for patients with S‐ICD treatment,^25^ similar to the outcome of MADIT RIT arm B and C. High rate cut‐off and delayed therapy programming were also associated in MADIT RIT with a 50% mortality reduction and no increase of syncope. The programming according to protocol was required as best standard, deviations have been documented and monitored throughout the study. Device patients were tracked with the remote monitoring system LATITUDE.

**TABLE 2:** Overview of programming options for defibrillator treatment

Defibrillator parameter	Long delay programming values	High rate cut‐off programming values
Defibrillator programming zones	3 zones	≥2 zones
Zone 1 Heart rate cut‐off	170 bpm	170 bpm
Zone 1 Duration before therapy	60 seconds	NA
Zone 1Defibrillator therapy	(ATP ‐ optional) + shock	Only monitoring of heart rhythm
Zone 2 Rate cut‐off	200 bpm	200 bpm
Zone 2 Duration before therapy	12 seconds	2.5 seconds or longer
Zone 2 Defibrillator therapy	Defibrillator therapy (shock +ATP [optional])	(ATP ‐ optional) + Shock
Zone 3 Heart rate cut‐off	250 bpm	>200 bpm (optional programming)
Zone 3 Duration before therapy	2.5 seconds or longer	(optional programming)
Zone 3 Defibrillator therapy	(ATP ‐ optional) + shock	(optional programming)
Programming S‐ICD scheme		
Heart Rate for conditional therapy zone		200 bpm
Therapy Shock Zone		250 bpm

Note

ATP, anti‐tachycardia pacing; bpm, beats per minute; S‐ICD, subcutaneous internal cardioverter defibrillator.

### 2.3 Follow‐up

Enrollment was open for 24 months, from July 2017 until July 2019. All participants remained in the study for a minimum of 12 months up to study close, ie, when the last participant completed the 12‐month visit.

After enrollment, patient follow‐up occurred every 6 months until the close‐out visit. Every other 6‐month visit could be replaced by phone follow‐up if the patient was connected to a home monitoring system and no standard of care in‐clinic visit was performed. During every visit, device assessments were performed according to standard of care. Any serious or nonserious adverse events (AEs) were recorded during follow‐up. The onset of life‐threatening arrhythmias, death, and HF events were documented for all device cohorts. Echocardiographic parameters and laboratory data were collected during the study period.

### 2.4 Primary endpoints

Primary endpoints of ICD and CRT‐D cohorts were the composite rate of the first appropriately treated VA (by ATP or shock) or life‐threatening symptoms associated with VA (defined as hemodynamic instability requiring treatment), whichever came first under MADIT Arm B (Intervention at high cut‐off rate: VT ≥200 bpm) or C (Intervention delay ≥60 second before therapy ≥170 bpm) programming conditions.^19^, ^20^ In the ND and PA cohorts, the primary endpoint was defined as all‐cause mortality.

### 2.5 Secondary endpoints

In the ICD and CRT‐D cohorts, the secondary endpoint was all‐cause mortality. In addition, HF events were regarded as the secondary endpoint in all the device cohorts. In the nondevice cohort, the rate of SCD was defined as the secondary endpoint.

### 2.6 Event adjudication

In order to ensure that data would be directly comparable with MADIT trials, this study had three event committees: Ventricular Event Committee (VEC), HF and Mortality Committee, and Electrocardiogram (ECG) Committee, which included independent experts from outside Japan who centrally assessed events relevant to the primary study endpoints (onset/treatment of all VA events, endpoint‐relevant events, all potential HF, and mortality events). The primary approach for VEC, HF, and mortality event adjudication was review of each event by two independent experts. If the results were discordant, that event was assigned to a third reviewer, who received identical information and completed the adjudication using the same submission process.

The VEC evaluated onset/treatment of all following VAs that may cause hemodynamic instability and impact patient management: all shock or anti‐tachycardia pacing (ATP) treatment events, all nonsustained events in the therapeutic range (≥200 bpm), and any event that is sustained for ≥30 seconds in the monitoring range (>170 bpm). The committee determined whether these events were relevant to the primary endpoints of the study. The relevant events include first appropriately treated VA by ATP or shock and life‐threatening symptoms associated with VA (defined as hemodynamic instability that requires treatment). To support VA event data collection in all device patients, the home monitoring system submitted reports on device programming and recorded electrograms of detected sustained/non‐sustained atrial/ventricular arrhythmias. All recorded VAs and treated arrhythmias were uploaded into the study database for adjudication and compared with study centers AE reporting.

Adjudication of HF events was based on signs and symptoms and needed to meet the following conditions: patients are admitted and discharged with a calendar date change (hospitalization) and receive new or increased decongestive HF regimen or inotropes with oral or parenteral medications during an in‐hospital stay (at least 1‐night), or patients are not hospitalized but receive IV decongestive therapy of one or more drugs, including diuretics, inotropes, or vasodilators, or other parenteral therapies. The HF and death Event Committee classified HF as a primary cause and an endpoint event if at least 2 of the following criteria were met: shortness of breath, weight gain (not induced by increased food intake), peripheral edema, pulmonary congestion or rales, pulmonary edema, pleural or pericardial effusion, hypotension, hypertensive crisis, dehydration, and rapid heart rate (>100 bpm at rest, either with sinus rhythm or AF). The committee adjudicated all death events and classified the cause of death in detail.

The ECG‐committee determined whether patients met ECG‐related criteria for study inclusion, exclusion, and assesses outcomes at the closeout visit: rhythm, intrinsic or paced QRS duration, PR and QT interval, and QRS morphology.

### 2.7 Statistical analysis

This study was designed to evaluate event rates in the Japanese patient population based on per treatment analysis and to compare them with historical data from landmark trials. The primary endpoint for each cohort was analyzed by a specific sample size calculation to reach 90% power. The sample size for each primary endpoint was estimated using exact binomial methods for comparison of a single proportion to a performance goal using a one‐sided significance level of 2.5%. (Table 3). Expected performance and performance goals for primary endpoints were sourced from previous studies. Performance measures were estimated based the following event rates, 5% of the ICD treated patients and 3% of the CRT‐D treated patients will present an appropriately treated first VA‐associated symptom (by ATP or shock) within 1 year (MADIT‐RIT)^20^; in the nondevice cohort, all‐cause mortality within 1 year was estimated to be 10% (MADIT‐II)^3^; and in the PA cohort, all‐cause mortality within 1 year was estimated to be 10% (CARE‐HF).^5^ Analysis of the primary and secondary endpoints on freedom from all‐cause mortality and first VA will be performed for each cohort using the Kaplan‐Meier curve for estimation of event free rates at 12/24‐months. Cohort event rates on VA, HF, and death will be descriptively compared with historical data. For best possible comparison between MADIT RIT and HINODE patient estimates will be further compared by subgroups within each cohort and to MADIT RIT outcomes using log‐rank tests. Separate propensity score (PS) match analyses will be performed to compare event rates for HV cohorts to MADIT RIT. For the HV cohort, ICD patients with CRT‐D indication (LVEF >35% and LBBB with QRS>130 ms or QRS>150 ms) will be removed, and for HV and ND cohorts’ patients with chronic AF will be removed prior to PS match to parallel MADIT RIT exclusion criteria. Remaining patients will be 1:1 matched to MADIT RIT arm B/C patients (novel ICD programming) on PS of select baseline characteristics with an exact match on pp indication. For reduction in standardized mean difference of characteristics, post‐PS match, or assessed model fit; a difference of less than 0.20 was desired. Baseline characteristics included those with high relevance toward the endpoint and availability in both studies. QRS width for MADIT RIT – ICD indicated that patients are not available, and BMI will be excluded from the HV match because of burden on model fit. Event rates will be re‐estimated in matched HINODE and MADIT RIT cohorts and compared using a log‐rank test stratified by the quintiles of the PS.

**TABLE 3:** Estimates for sample size per cohort

Cohort	Primary endpoint measurement (0 to 12 months)	Performance goal hypotheses^a^	Expected performance^b^	Power	Number of patients needed for Analysis
ICD	VA associated symptoms free‐rate	H_0_: Rate ≤85.0% H_A_: Rate >85.0%	95.0%	90%	93
CRT‐D	VA associated symptoms free‐rate	H_0_: Rate ≤87.0% H_A_: Rate >87.0%	97.0%	90%	76
No‐device	All‐cause mortality free‐rate (survival rate)	H_0_: Rate ≤80.0% H_A_: Rate >80.0%	90.0%	90%	137
Pacing	All‐cause mortality free‐rate (survival rate)	H_0_: Rate ≤80.0% H_A_: Rate >80.0%	90.0%	90%	137

^a^ Hypothesis test based on a one‐sided alpha level of 0.025; clinically accepted delta of 10%, subtracted from expected performance.

^b^ Determined from prior comparable studies.

Additionally, the rate of SCD for the ICD or CRT‐D indicated nondevice cohort will be compared with the rate of appropriately treated VAs or SCD in the ICD and CRT‐D cohorts. Exploratory analyses for the pooled ICD and CRT‐D cohorts versus nondevice cohorts will be performed using the propensity score matching method to compare the all‐cause mortality rate and the composite rate of HF events between the cohorts.

Characteristics for ICD, CRT‐D, and ND cohorts will be compared using Tukey's test for continuous variables and chi‐squared test with Bonferroni correction for categorical variables. A significance level of 0.05 will be used.

## 3 DISCUSSION

The present study aims to evaluate the risk of SCD and HF events in Japanese patients using the risk stratification in the ESC guideline.^6^ Although indication of primary defibrillator therapy was clearly described in the Japanese guideline published in 2011,^22^ the proportion of patients receiving a defibrillator device as primary prevention remains lower than in western countries.^2^, ^9^ The CHART‐2 study reported that only 30.4% of enrolled Japanese HF patients that satisfied the Class I indication and 6.6% of the patients that satisfied Class IIa indication received ICD therapy.^10^ Previous registries reported lower rates of SCD in the Japanese population,^7^, ^8^ which may have impacted decision making for implant of primary defibrillator device. Nevertheless, rate of SCD satisfying Class I and IIa indications in the CHART‐2 study was higher than previous registries.^7^, ^8^ Additionally, the Seattle Proportional Risk Model (a risk model calculated from western countries) also predicts future events in Japanese patients.^23^ Thus, SCD event rates in Japan may be higher than assumed, and Japanese HF patients share similar risk factors with patients in western countries. To improve patient management, a large registry is warranted to clarify the incidence and therapeutic impact of implantable devices in Japanese HF patients.

The MADIT‐RIT trial enrolled 68 Japanese primary prevention patients with ICD and CRT‐D therapy. Analysis of this MADIT RIT subcohort presented similar rates of supraventricular, ventricular arrhythmias, appropriate ICD therapy, and mortality, but they had a higher rate of inappropriate ICD therapy, especially in ischemic patients, as compared to non‐Japanese patients.^24^ The defibrillator programming of HINODE ICD and CRT‐D devices followed arm B and C of the MADIT RIT study which presented reductions in inappropriate therapy and all‐cause mortality.^21^ This is the best possible programming standard for defibrillator treatment in primary prevention cohorts.^20^

The Heart Failure Indication and Sudden Cardiac Death Prevention Trial Japan includes diverse patient history across multiple treatment groups and will provide the opportunity to analyze the effectiveness of different treatment strategies by comparing Japanese primary prevention patient event rates of: first appropriately treated VA, all‐cause mortality, and HF with event rates found in similar cohorts in MADIT trials.^3^, ^4^, ^21^ The enrolled patient data will allow analysis of event rates for the treatment cohorts and multivariable analysis may help to identify alternative treatment options. The event rates of the ICD and CRT‐D cohort, as well as the combined HV cohort, will be compared with MADIT RIT event data on primary prevention patients treated by ICD and CRT‐D therapy especially from Arm B and C with analog device programming. The ND cohort is enrolling ICD and CRT‐D indicated subjects and will give more insight on potential high‐risk subgroups for mortality, HF events or device upgrades. The PA cohort is focusing on CRT‐P and CRT‐D indicated patients who are treated by single, dual, or triple chamber pacing device. ND and PA cohorts may give guidance on optimized strategies for device treatment decision.

## 4 CONCLUSIONS

The HINODE study is designed to evaluate effectiveness of current approaches to HF treatment and SCD prevention in Japanese patients. This study will allow comparison of current Japanese clinical care to reports from western countries. Results of the HINODE study may help to identify at risk Japanese populations that would benefit from alternative therapeutic approaches.

## CONFLICT OF INTEREST

Yamasaki H. has no conflict to report. Ando, K. received lecture fees from Japan Lifeline Co., Ltd, Terumo Co., Ltd., Bristol‐Myers Squibb Co., Ltd, Medtronic Japan Co. Ltd., Biotronik Japan, Bayer Co., Ltd. and Boston Scientific Japan. Ikeda, T. received scholarship funds or donations scholarship funds from Medtronic Japan Co., Ltd., Japan Lifeline Co., Ltd., and Daiichi Sankyo Co., Ltd. and remuneration from Ono Pharmaceutical Co., Ltd., Bayer Co., Ltd., and Bristol‐Myers Squibb Co., Ltd.. Mitsuhashi, T. received lecture fees from Medtronic Japan Co., Ltd., and Abbott Japan. Murohara, T. has no conflict of interest. Nishii, N. belonged to the endowed department by Medtronic Japan Co., Ltd. and received the lecture fee from Medtronic Japan Co., Ltd., and Boston Scientific Japan. Nogami, A. received honoraria from Johnson & Johnson, Boehringer‐Ingelheim, Daiichi‐Sankyo Co., Ltd., and Abbott Japan and an endowment from Medtronic Japan Co., Ltd., and DVx Co., Ltd.. Sakata Y. received a scholarship fund granted from Boston Scientific Japan. Shimizu, W. has no conflict of interest. Simon T., Beaudoint C., and Kayser T. are employees at Boston Scientific. Kutyifa, V. received research grants from Boston Scientific, ZOLL, Biotronik, Spire Inc, consultant fees from Biotronik, and ZOLL. Aonuma, K. received speaker honoraria from Abbott Japan, Boehringer‐Ingelheim, Daiichi‐Sankyo Co., Ltd., Boston Scientific Japan and belonged to an endowment department by Abbott Japan.

## Data Availability

The data underlying this article will be shared upon reasonable request to the corresponding author.
